# The dysregulation of PARP9 expression is linked to apoptosis and DNA damage in gastric cancer cells

**DOI:** 10.1371/journal.pone.0316476

**Published:** 2024-12-31

**Authors:** Yating Li, Xing Wang, Xiaolong Liu, Xiangjie Li, Jianling Zhang, Yulan Li

**Affiliations:** 1 The First School of Clinical Medical, Lanzhou University, Lanzhou, Gansu, P.R. China; 2 The Second Clinical Medical College, Lanzhou University, Lanzhou, Gansu, P.R. China; 3 General Surgery Ward 5, The First Hospital of Lanzhou University, Lanzhou, Gansu, P.R. China; Università degli Studi della Campania, ITALY

## Abstract

**Background:**

Gastric cancer (GC) is a highly malignant gastrointestinal tumor characterized by difficult early diagnosis and poor prognosis. Therefore, it is imperative to explore potential therapeutic targets for gastric cancer. PARP9 is abnormally expressed in a variety of tumors and is associated with tumor cell apoptosis and DNA damage. However, its relationship with GC has not been fully studied.

**Methods:**

The expression and prognostic significance of PARP9 in gastric cancer (GC) were examined using bioinformatics approaches. Cell lines with either knockdown or overexpression of PARP9 were established through lentiviral transduction, and the role of PARP9 in the malignant phenotypes of GC cells was validated via CCK8 assays, wound healing assays, clonogenic assays, and Transwell migration experiments. Finally, alterations in downstream targets and signaling pathways following changes in PARP9 expression were analyzed through RNA sequencing.

**Results:**

PARP9 is highly expressed in GC tissues and is associated with poor prognosis. PARP9 knockdown can significantly inhibit the proliferation, invasion and migration of GC cells, and increase the apoptosis and DNA damage of GC cells. The therapeutic process of PARP9 in GC may be realized by synergistic interaction with SOX6 through MAPK signaling pathway.

**Conclusions:**

Our study reveals a potential link between PARP9 and GC, providing a new target for the treatment of GC.

## 1. Introduction

GC ranks as the fifth most prevalent malignancy globally and is the fourth leading cause of cancer-related mortality [1]. Despite a general decline in global incidence rates, there has not been a corresponding significant reduction in mortality [2]. In 2020, approximately two-thirds of newly diagnosed cases of stomach cancer were reported in East and Southeast Asia [3]. Helicobacter pylori infection is currently recognized as the primary etiological factor for GC, with China and South Korea exhibiting the highest infection and prevalence rates [4, 5]. The incidence of GC predominantly affects older adults, typically peaking during the seventh and eighth decades of life. However, it is often diagnosed at advanced stages, severely limiting treatment options. Once patients reach middle to late-stage disease, their prognosis becomes exceedingly poor, with five-year survival rates generally ranging from 30% to 35% [6] Although novel therapeutic approaches—including immune checkpoint inhibitors, cellular immunotherapy, and cancer vaccines—have made substantial advancements, their efficacy remains limited for patients with advanced disease. Therefore, elucidating the mechanisms underlying the occurrence and progression of gastric cancer remains crucial for reducing local recurrence and metastasis while identifying specific molecular markers that could enhance GC therapy.

Cells utilize post-translational modifications to fine-tune signaling pathways, encompassing DNA repair, genomic stability, and programmed cell death in response to both external and internal stimuli [7]. A prominent modification is ADP-ribosylation (ADPr), which is conserved across all organisms and covalently attaches to amino acid residues of proteins as well as nucleic acids [8]. In humans, this modification is primarily catalyzed by 17 enzymes belonging to the PARP family [9]. PARP serves as a crucial DNA repair enzyme that plays an essential role in the restoration of DNA integrity and apoptosis [10]. Targeting Poly(ADP-ribose) polymerase (PARP) enzymes has emerged as a promising therapeutic strategy for cancer treatment [11]. A PARP inhibitor (PARPi), specifically designed to leverage synthetic lethality through targeting ADP-ribose, was the first clinically approved drug of its kind [12]. However, resistance to PARPi is widespread in clinical practice; more than 40% of patients with BRCA1/2 deficiencies exhibit inadequate responses to this therapy. Additionally, many patients develop resistance following extended oral administration of PARPi [13].

While significant attention has been devoted to PARP1 and PARP2, comparatively less focus has been directed towards PARP9. As a member of the PARPs family sharing catalytic activity with PARP13 [14], previous investigations have demonstrated that PARP9 displays aberrant expression patterns in various tumors including breast, prostate, and cervical cancers; it is also associated with recurrent drug resistance within these malignancies [15–17]. Nonetheless, the relationship between PARP9 and gastric cancer remains inadequately characterized. By synthesizing existing knowledge on the role of PARP9 in other tumor types and elucidating its function within gastric cancer may reveal novel therapeutic targets for managing this disease.

In this study, we initially identified that PARP9 is highly expressed in GC, and its elevated expression correlates with poor prognosis in GC patients. Furthermore, the overexpression of PARP9 enhances the malignant biological behaviors of GC both in vivo and in vitro. Additionally, PARP9 interacts with SOX6 to promote resistance of GC cells to apoptosis and DNA damage through the MAPK signaling pathway. In summary, this study elucidates the regulatory mechanism by which PARP9 modulates SOX6 in GC and presents a novel therapeutic target.

## 2. Materials and methods

### 2.1. Bioinformatics

GEPIA (Gene Expression Profiling Interactive Analysis) and UALCAN (The University of ALabama at Birmingham CANcer data) The analysis Portal online database was used to examine the expression of PARP9 in TCGA+GTEx and TCGA-STAD respectively. Download the GSE118916 dataset in the Gene Expression Omnibus (GEO) to observe the expression of PAPR9 in paired tissues. The Kaplan-Meier plotter (http://kmplot.com/analysis/) was utilized to examine the relationship between PARP9 expression and OS (Overall Survival) of GC patients. The ROC (Receiver Operating Characteristic) was employed to evaluate the predictive accuracy of PARP9 in GC.

### 2.2. Ethics statement

TCGA and GEO belong to public databases. The patients involved in the database have obtained ethical approval. Users can download relevant data for free for research and publish relevant articles. Our study is based on open source data, so there are no ethical issues and other conflicts of interest.

### 2.3. Cell culture

Normal gastric epithelial cells GES-1 and three GC cell lines (HGC-27, AGS and MKN-45) were purchased from Peking Union Medical College. All cells were cultured in RPMI 1640 (Gibco, USA) with 10% FBS and 1% penicillin and in a 37°C, 5% CO2, 95% air incubator (Thermo, USA).

### 2.4. Transfection of lentiviruses and plasmids

The lentiviruses for knockdown and overexpression were engineered and synthesized by Generalbiol in Anhui Province, China. The transfection procedures were conducted in accordance with the instructions provided by the lentivirus company. The plasmids were designed and fabricated by Beijing Sino Biological Inc. (Catalogue Number: NM_001145811.1). The transfection process utilized Lipofectamine 2000 (Thermo Fisher, USA), and was performed following the stepwise instructions furnished by the reagent provider.

### 2.5. Real time PCR

RNA was extracted from tissues and cells using TRIzol reagent, and cDNA was synthesized. The kit used was from Vazyme biotech (China). According to the instructions provided by the reagent supplier, the samples were added, and real-time fluorescence quantitative PCR was performed. The relative expression was calculated using 2-ΔΔCt, and normalization was performed using β-actin. The sequences of the primer pairs are shown below: PARP9 forward, 5’-GGCCTCGGTGGATGGAATG-3’, reverse, 5’-GCAAACTAACCCGGATAGTCTCT-3’; MYH4 forward, 5’-GCTGCTTATCTGACAAGTCTGAA-3’, reverse, 5’- GGCCTTTGGTTACGAACTCATT -3’; SLC7A11 forward, 5’- TCTCCAAAGGAGGTTACCTGC-3’, reverse, 5’- AGACTCCCCTCAGTAAAGTGAC-3’; RYR2 forward, 5’- ACAACAGAAGCTATGCTTGGC-3’, reverse, 5’-GAGGAGTGTTCGATGACCACC-3’; INHBE forward, 5’- ATCTTCCGATGGGGACCAAG-3’, reverse, 5’-GCAAACTAACCCGGATAGTCTCT-3’; SYTL5 forward, 5’- ATGATCCTGGGCGTCCTAAAG-3’, reverse, 5’- TCCCACTTCTACGTTTTGCTTC-3’; MATN2 forward, 5’- GATCCTCGGACAGATCGTCCT-3’, reverse, 5’- CTGCCCGCTTGTTCTCACA-3’; SLC46A3 forward, 5’- CGGTCCACTGACAACGCAATA-3’, reverse, 5’- GGGAATTTTCGTCCGTAGTGAT-3’; RIMKLB forward, 5’- GTTTTTGACAGATCGTCGCATC-3’, reverse, 5’- AGTTCCTCCTCACAACATTTGG-3’; PTPRO forward, 5’- CCCAATGTGGTAGTGATCTCCG-3’, reverse, 5’- AGCTTTCCATCCCTCTCTAAGG-3’; SOX6 forward, 5’- GGATGCAATGACCCAGGATTT-3’, reverse, 5’- TGAATGGTACTGACAAGTGTTGG-3’; GAPDH forward, 5’-GGAGCGAGATCCCTCCAAAAT-3’, reverse, 5’-GCTGTTGTCATACTTCTCATGGG-3’.

### 2.6. Western blot

Add RIPA lysate (Beyotime, China) and PMSF (Beyotime, China) to the cells at a ratio of 100∶1, and replace the PMSF with a phosphatase inhibitor (Biosharp, China) for phosphorylated proteins. Sonicate the cells and centrifuge at 12,000g for 20 minutes. Collect the supernatant. Measure the protein concentration using the BCA assay kit (Beyotime, China). Separate the denatured proteins using 10% SDS-PAGE and transfer them to a PVDF membrane. Block the membrane with rapid blocking solution, incubate the primary antibody overnight, and wash before using an enzyme-labeled secondary antibody to bind to it. Finally, visualize the spots using ECL super-sensitive luminescence liquid (Beyotime, China). The used primary antibodies include PARP9 and SOX6 (Abcam, USA), Caspase-3, BCL-2, Bax, ERK1/2, p-ERK1/2, p38, and p-p38 (CST, USA), XRCC1, XRCC2, and PALB2 (Proteintech, China). All raw data of Western blot in this study were uploaded to supplement file S1 File.

### 2.7. Wound healing

GC cells were inoculated in 6-well plates and cultured to a fusion degree of more than 90%. The wound area was measured and cell mobility was calculated using an inverted microscope (Olympus, Japan) after 48 h.

### 2.8. Transwell experiment

The migration ability of GC cells was determined by Transwell assay. 5×104 cells were suspended with basal culture and inoculated into the upper layer of Transwell chamber (Corning, USA), followed by the addition of 800ul in the lower layer. Put in the incubator for 24h, take out the chamber on the second day, gently wipe the upper chamber cells with a cotton swab, wash and fix the methanol, stain with crystal violet, and take photos.

### 2.9. Colony formation assay

The cells were inoculated in the six-well plate with 1 × 103 cells per well and cultured until the cell colony was formed. After washing, the methanol was fixed for 30 minutes, and the crystal violet was dyed at room temperature for 30 minutes.

### 2.10. Flow cytometry

In the apoptosis experiment, cells were collected, the cells were suspended with a buffer, and then 5μl Annexin V-FITC and 10μl PI were added to the flow tube (Beyotime, China). After incubation at room temperature without light for 30min, the samples were detected by flow cytometry (Beckman, USA).

### 2.11. Comet experiments

A single-cell suspension is prepared, added to an agarose gel, the cells are subsequently cleaved, the slide is placed in a horizontal electrophoresis tank, and an electrophoresis buffer is added so that the double-strand unhelix is single strand, and the damaged DNA fragments migrate to the anode under specific voltage and current conditions. After electrophoresis, the slides were treated with neutralization buffer, dyed and photographed.

### 2.12. RNA sequencing and data analysis

RNA sequencing and data analysis were conducted by Tsingke Technology(Beijing, China). Total RNA was extracted from AGS cells that were either PARP9 knockout or non-knockout using TRIzol Reagent (Takara, Japan). After the sample was determined to be qualified, standard RNA sequencing library was prepared, and the sequencing was performed using Novaseq 6000 sequencer (Illumina). The subsequent data analysis was completed by Tsingke Technology.

### 2.13. Co-immunoprecipitation

Cells were cultured in 10cm dishes (cell number at least 1*107), and when the cells were fully grown, they were collected, and AGS cells were lysed with a lysis buffer containing proteinase inhibitors. The cells were lysed on ice for 10 minutes. The lysate was centrifuged at 14,000 rpm for 15 minutes to collect the supernatant. The target antibody was incubated with the protein sample together, and incubated overnight at 4°C. Then, it was mixed with previously treated Protein A/G beads (ACE, China) at room temperature for 1 hour. The complexes bound to Protein A/G were washed, and then the protein blot was performed.

### 2.14. Statistical analyses

Statistical analyses were performed using SPSS (version 26.0) and R (version 4.2.1). Numerical data were analyzed using one-way analysis of variance (ANOVA) or Student’s t-test (two-tailed) for statistical significance, and expressed as mean±standard error of the mean (SEM). Two-sided t-tests were used for comparisons between groups, and ANOVA was used for comparisons among multiple groups. Survival analysis was performed using the Kaplan-Meier method, and group differences were analyzed using the Log-rank test. P < 0.05 was considered statistically significant.

## 3. Result

### 3.1. High expression of PARP9 in gastric cancer correlates with poor prognosis

**Fig 1:**
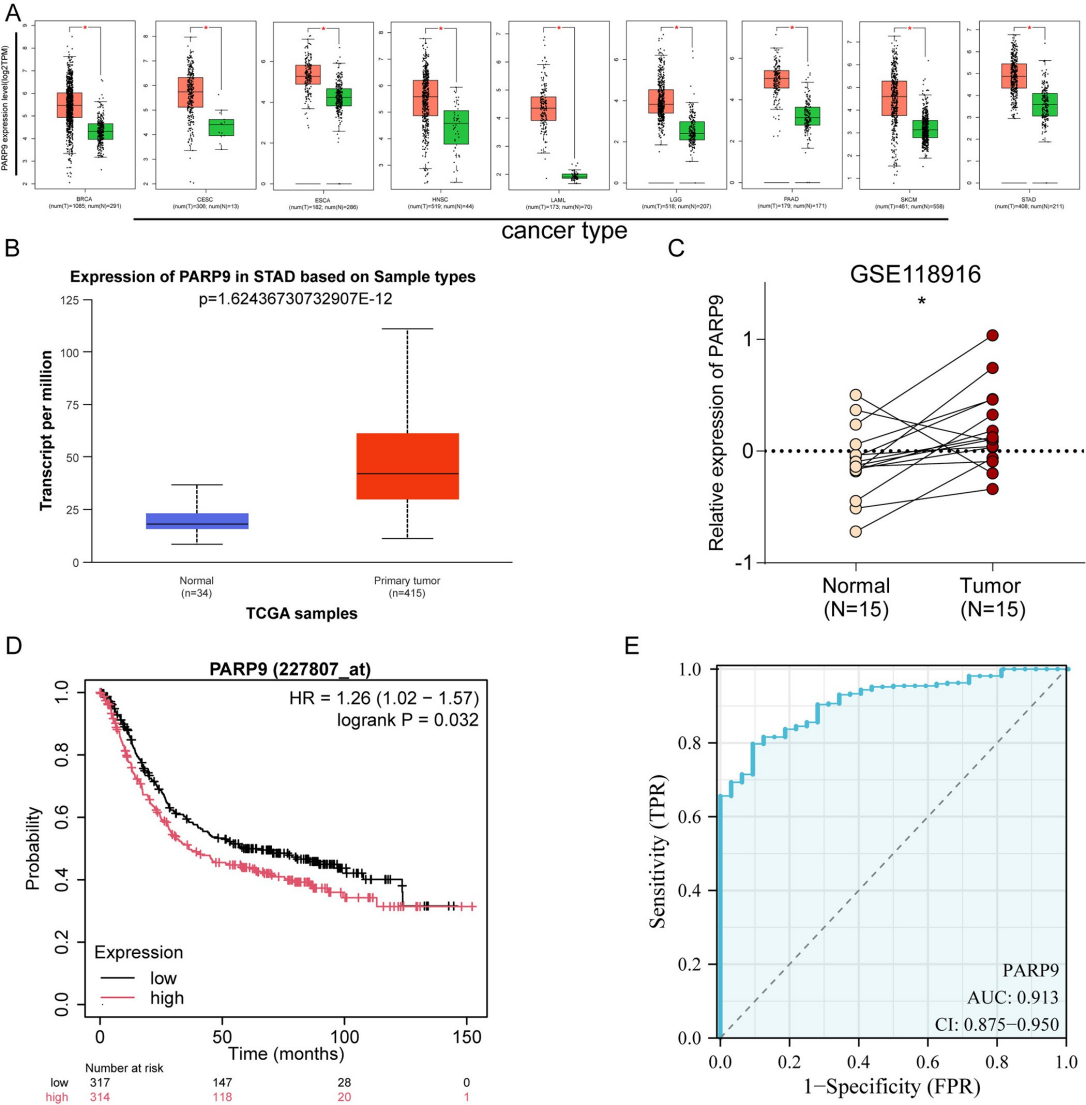
High expression of PARP9 is associated with poor prognosis in gastric cancer (GC). (A) A pan-cancer analysis of PARP9 was conducted using the GEPIA online database. (B) The Ualcan database was utilized to assess the expression of PARP9 in unpaired samples from TCGA. (C) The mRNA expression levels of PARP9 in GC and normal samples were evaluated using GSE118916. (D) Kaplan-Meier analysis indicated that high PARP9 expression correlated with poorer overall survival compared to low PARP9 levels in GC patients. (E) Receiver Operating Characteristic (ROC) curve analysis was performed to evaluate the predictive accuracy of PARP9 as a biomarker. *p < 0.05.

To investigate the role of PARP9 in GC, we initially assessed its expression across various malignant tumors using the online database GEPIA. The results indicated that PARP9 expression was significantly elevated in several malignancies, including gastric cancer, breast cancer, cervical squamous cell carcinoma, and cervical adenocarcinoma (Fig 1A). Further analysis through the Ualcan database corroborated these findings from TCGA data, revealing increased levels of PARP9 in gastric cancer (p = 1.6e-12) (Fig 1B). Additionally, examination of paired data from the GEO database demonstrated that PARP9 expression in GC tissues within GSE118916 was markedly higher than that observed in normal tissues(p = 0.032) (Fig 1C). Kaplan-Meier survival analysis revealed that patients exhibiting high levels of PARP9 had a poorer prognosis (HR = 1.26 [1.02–1.57], log-rank p = 0.032) (Fig 1D). Finally, ROC curve analysis confirmed that PARP9 exhibited high accuracy for predicting prognosis among gastric cancer patients (AUC = 0.913,CI: 0.875–0.950) (Fig 1E).

### 3.2. Knockdown of PARP9 inhibits malignant biological characteristics of gastric cancer cells

**Fig 2:**
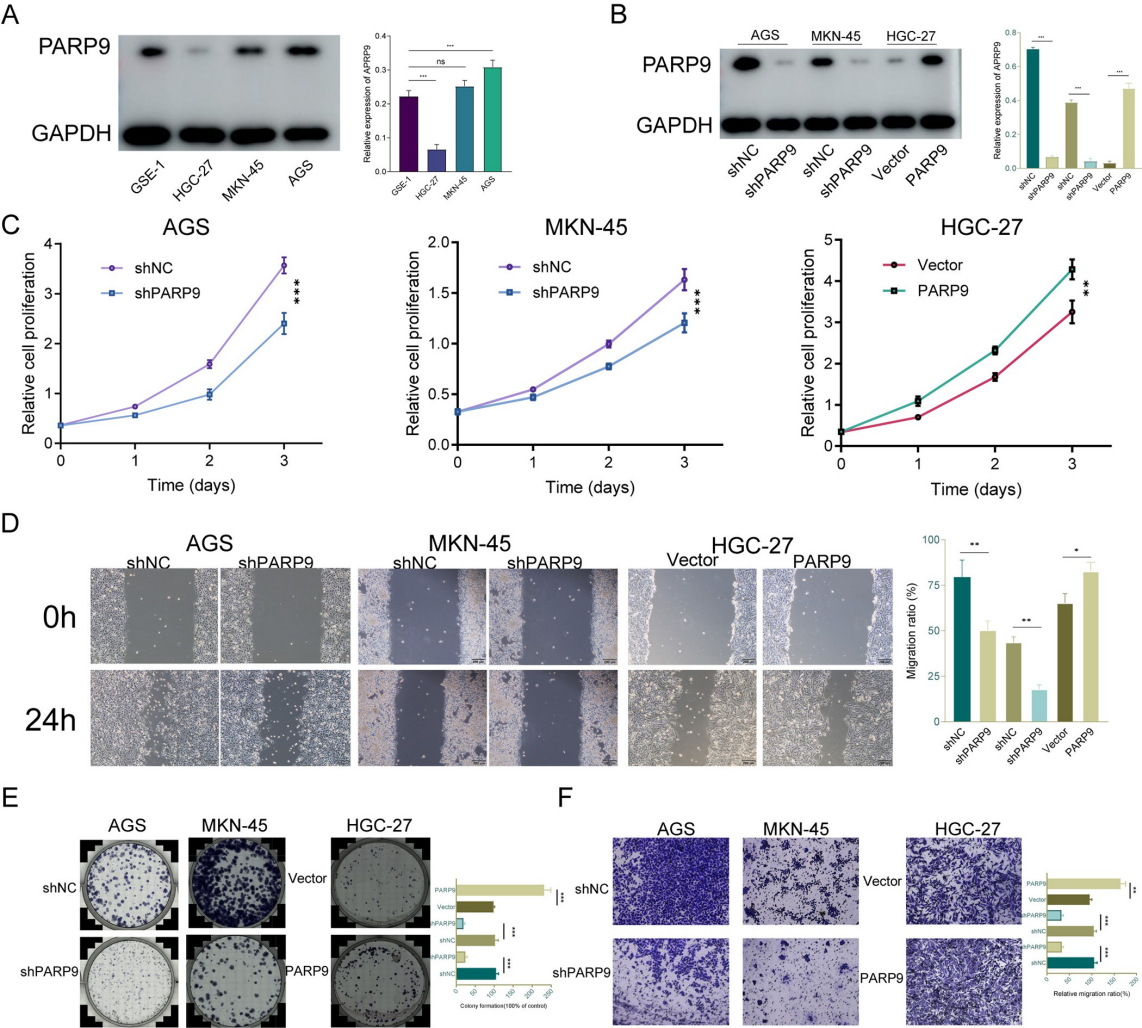
PARP9 knockdown can inhibit the malignant biological behavior of gastric cancer cells. (A) The expression of PARP9 in normal gastric epithelial cells and three GC cell lines was examined by western blot. (B) The expression of PARP9 in normal gastric epithelial cells and three GC cell lines was examined by western blot. (C) CCK8 proliferation assay to investigate the effect of PARP9 changes on the proliferation ability of GC cells. (D) Wound healing experiment to investigate the healing rate of three GC cells under the influence of PARP9 after 24 hours. (E) Monoclonal formation assay to examine changes in proliferation and population-dependent ability of GC cells after altered PARP. (F) Transwell experiment verified the change of invasion and migration ability of GC cells after PARP9 change.Bar = 200um. *p < 0.05, **p < 0.01, ***p < 0.001.

To investigate the role of PARP9 in GC development, we selected AGS and MKN-45 cell lines with relatively high expression levels, along with HGC-27 cells exhibiting lower expression (Fig 2A). Stable transfected cell lines for PARP9 were constructed, and transfection efficiency was verified via Western blot analysis. A series of in vitro loss-of-function and gain-of-function studies regarding PARP9 were conducted (Fig 2B). CCK8 assays demonstrated a significant decrease in cell viability following downregulation of PARP9, contrasting sharply with the marked increase in GC cell viability observed after overexpression of PARP9 (Fig 2C). To assess the impact of PARP9 on GC cell migration, wound healing experiments were performed across three GC cell lines,results indicated that the healing rate was significantly impaired post-PARP9 knockdown, while it was notably enhanced following overexpression (Fig 2D). Colony assays further confirmed that proliferation rates of GC cells increased upon overexpression of PARP9, whereas knockdown resulted in decreased proliferation (Fig 2E). Finally, Transwell assays were conducted to evaluate changes in invasion and migration capabilities among GC cells. The invasive and migratory abilities were significantly augmented following overexpression of PARP9 but diminished after knockdown (Fig 2F).

### 3.3. Effects of PARP9 on apoptosis and DNA damage in gastric cancer cells

**Fig 3:**
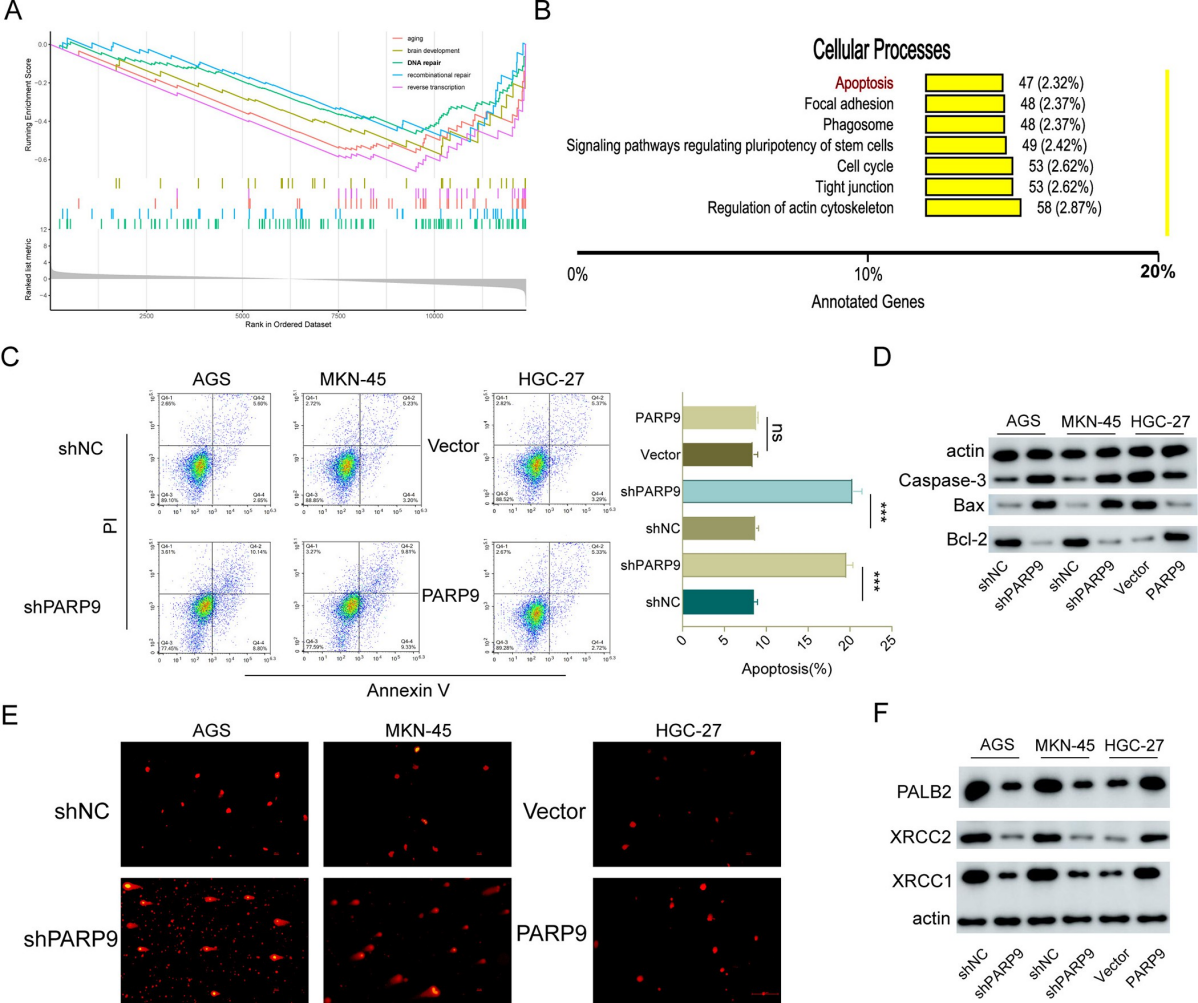
PARP9 knockdown promotes apoptosis and DNA damage. (A) Gene Set Enrichment Analysis (GSEA) was conducted to identify the major enriched pathways of differentially expressed genes following PARP9 downregulation. (B) Significant alterations in cellular processes of gastric cancer (GC) were observed after changes in PARP9 expression. (C) The apoptosis rate of GC cells significantly increased following PARP9 knockdown. (D) Western blot analysis was performed to assess the expression levels of Caspase-3, Bax, and Bcl-2 after alteration of PARP9. (E) DNA damage in GC cells significantly increased post-PARP9 knockdown as determined by comet assay. (F) The anti-DNA damage capacity of GC cells decreased markedly following reduction of PARP9 expression. Bar = 200um.***p < 0.001.

To investigate the influence of changes in PARP9 expression on GC, we compared AGS normal group cells with those subjected to PARP9 knockdown using RNA-sequencing. GSEA analysis revealed that differentially expressed genes were primarily enriched in pathways related to aging, brain development, and DNA repair, showing a negative correlation with these enrichment pathways as PARP9 levels decreased (Fig 3A). Subsequent analysis of the effects of PARP9 on cellular processes indicated that alterations were predominantly focused on the cell cycle and apoptosis (Fig 3B). Integrating results from RNA sequencing, we first examined the relationship between PARP9 and apoptosis; notably, the apoptotic rate significantly increased in AGS and MKN-45 cells following PARP9 knockdown. Specifically, the apoptotic rates rose from 5.60% ± 2.65% to 10.14% ± 8.80% for AGS cells and from 5.23% ± 3.20% to 9.81% ± 9.33% for MKN-45 cells, while no significant change was observed in HGC-27 cells after overexpression of PARP9 (Fig 3C). Western blot analyses corroborated this trend: following PARP9 knockdown, expressions of Caspase-3 and Bax were upregulated while Bcl-2 expression decreased; conversely, an opposite trend was noted upon overexpression of PARP9 (Fig 3D). In comet assays assessing DNA damage, a pronounced tailing phenomenon was evident when PARP9 expression was reduced—indicative of increased DNA damage—whereas no significant changes occurred upon overexpression (Fig 3E). Additionally, Western blot results demonstrated that expressions of three anti-DNA damage genes—PALB2, XRCC2, and XRCC1—decreased significantly after knocking down PARP9 but increased following its overexpression; this indicates that GC cells’ ability to counteract DNA damage diminished with reduced levels of PARP9 but improved with its overexpression (Fig 3F).

### 3.4. PARP9 influences gastric cancer progression through the MAPK pathway

**Fig 4:**
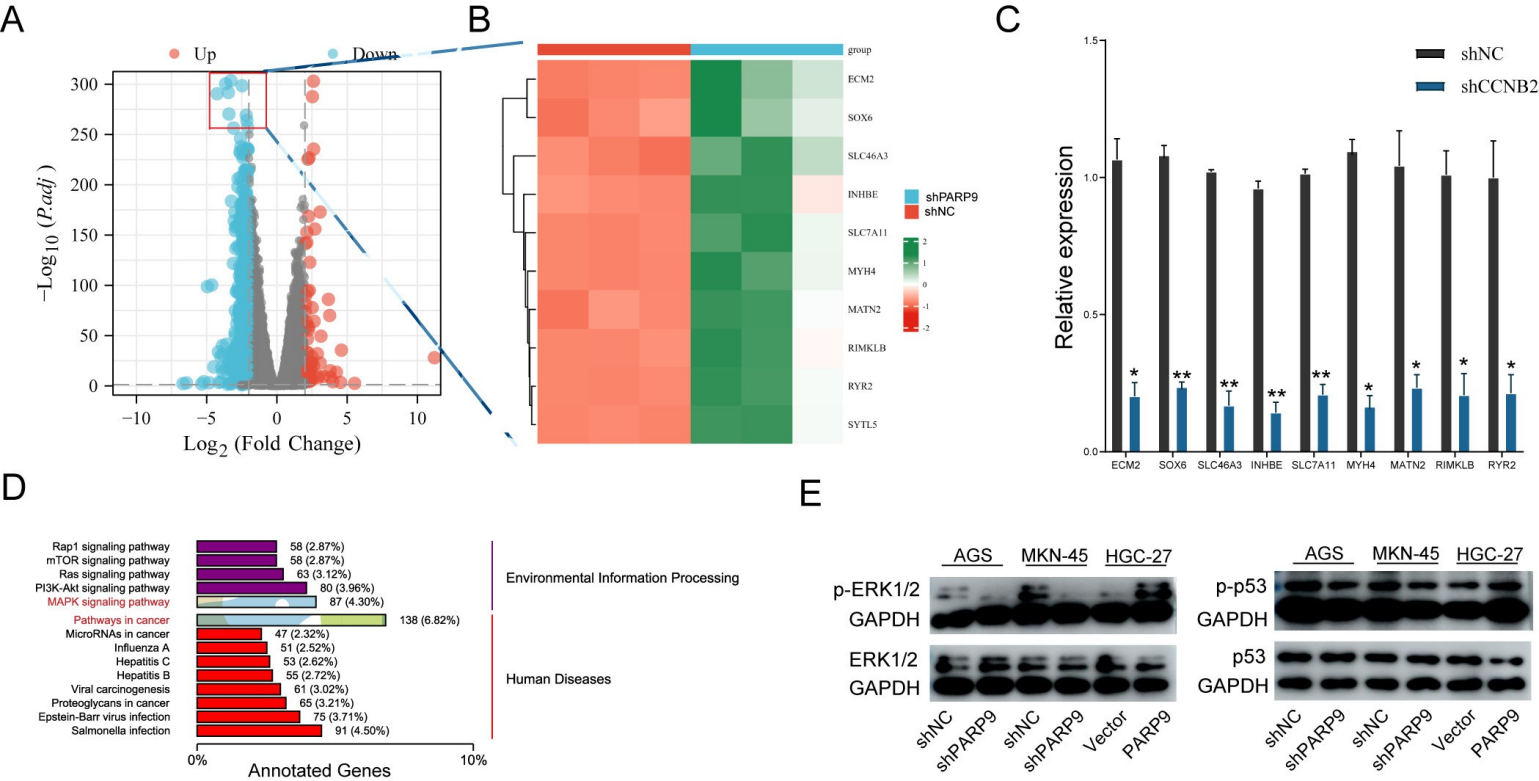
PARP9 exerts its effects through the MAPK signaling pathway. (A) Volcano plot of differentially expressed mRNA. (B) Heat maps depicting the top ten genes with the highest reduction ratios among the differential genes. (C) Quantitative reverse transcription PCR (qRT-PCR) validated the ten genes exhibiting the greatest reduction ratios. (D) Enrichment pathways of differentially expressed genes were analyzed using KEGG analysis. (E) Western blot analysis was performed to examine changes in ERK1/2 and p53 expression following alterations in PARP9 levels. *p < 0.05, **p < 0.01.

To further investigate the role of PARP9 in GC, a volcano plot was generated based on RNA sequencing results, revealing a total of 5,024 differentially expressed genes, comprising 1,788 upregulated and 3,236 downregulated genes (Fig 4A). A heatmap was constructed by analyzing the top ten genes with the most significant downregulation ratios; expression levels of these genes were markedly decreased (Fig 4B). qRT-PCR validated that the expression of these ten genes was also reduced (Fig 4C). Further analysis indicated that in human diseases, differentially expressed genes were predominantly associated with cancer. In terms of Environmental Information Processing, differential gene enrichment primarily focused on the MAPK signaling pathway and PI3K/AKT signaling pathway (Fig 4D). Finally, based on sequencing results, we examined changes in key proteins ERK1/2 and p38 within the MAPK pathway following alterations in PARP9 levels using Western blot analysis. When PARP9 expression was reduced, no significant changes were observed for ERK1/2 and p38; however, expressions of phosphorylated ERK1/2 and p38 decreased. Conversely, when PARP9 levels increased, both ERK1/2 and p38 showed diminished changes while phosphorylated forms exhibited elevated expressions (Fig 4E).

### 3.5. Interaction between PARP9 and SOX6

**Fig 5:**
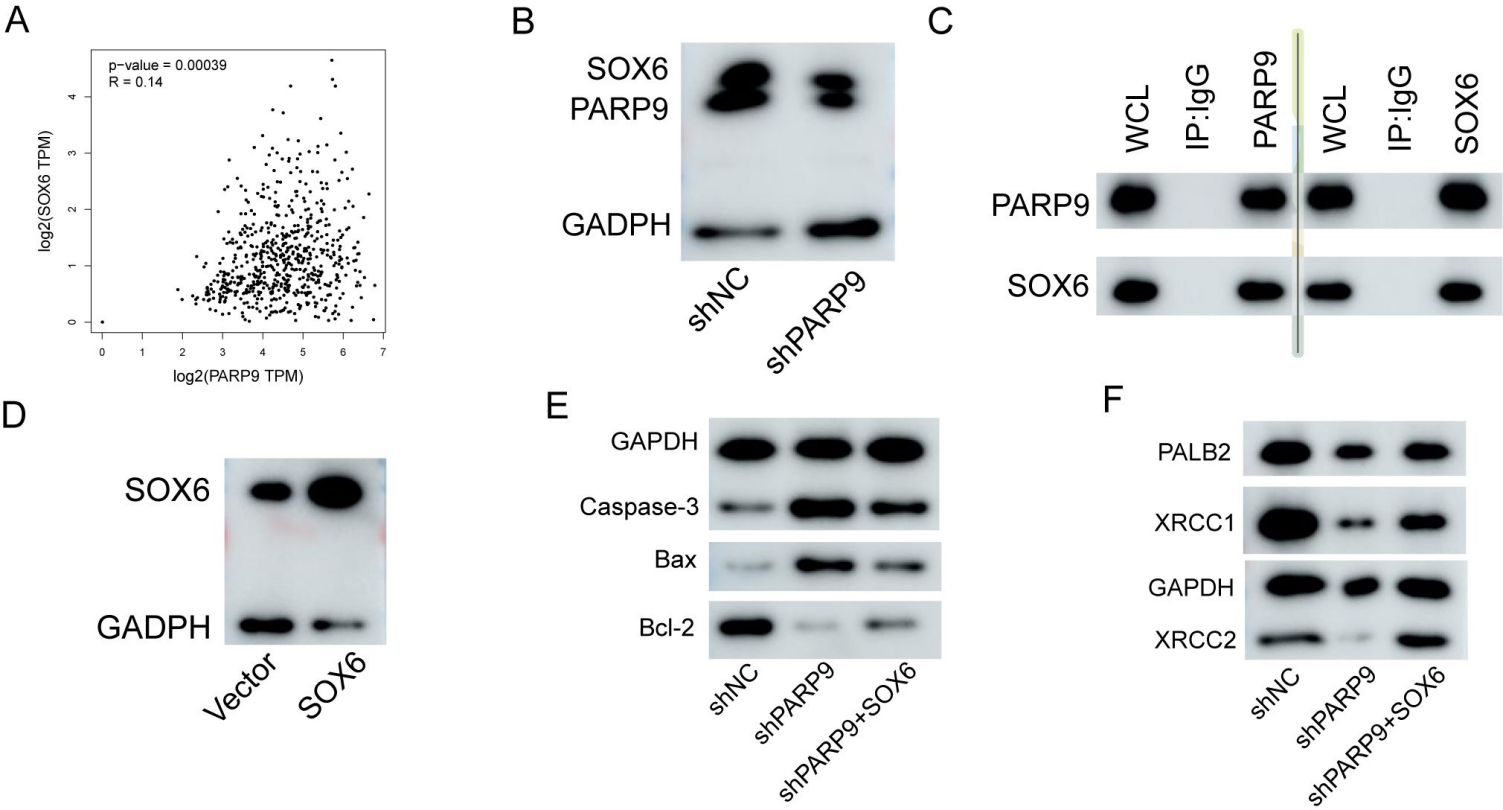
Over expression of SOX6 attenuates apoptosis and DNA damage in AGS cells. (A) The correlation between PARP9 and SOX6 was validated using an online database. (B) At the protein level, it was confirmed that SOX6 levels decreased concomitantly with diminishing PARP9 expression. (C) Co-immunoprecipitation (COIP) confirms the interaction between PARP9 and SOX6. (D) The overexpression of SOX6 was verified through Western blot analysis. (E) Changes in apoptotic proteins in AGS cells were assessed via a response experiment. (F) The response experiment also confirmed alterations in anti-DNA damage proteins within AGS cells.

To further investigate the pathogenic mechanism of PARP9, we examined the correlation between PARP9 and the top ten genes using the GEPIA database. The results indicated a significant correlation between PARP9 and SOX6 (Fig 5A). Western blot analysis revealed that when PARP9 expression was decreased, SOX6 expression also diminished (Fig 5B). To explore the potential interaction between PARP9 and SOX6, an endogenous immunoprecipitation assay confirmed their interaction (Fig 5C). Subsequently, to assess the effect of SOX6 overexpression on PARP9 knockdown in gastric cancer cells, we first transfected AGS cells with a plasmid encoding SOX6 and verified overexpression efficiency via Western blot analysis (Fig 5D). Finally, by overexpressing SOX6 in AGS cells with knocked-down PARP9 expression, we observed a significant decrease in apoptosis index (Fig 5E) as well as enhanced anti-DNA damage capability (Fig 5F).

## 4. Discussion

GC is a highly prevalent gastrointestinal tumor characterized by poor prognosis, as well as high rates of recurrence and metastasis [18]. Therefore, it is essential to investigate the complex mechanisms underlying GC and identify effective therapeutic targets to impede its progression. Improvements in molecular analysis open new prospects for personalized therapy, targeting HER2, Claudine, FGFR, and other changes in molecular-matched therapy can significantly improve clinical outcomes in patients with advanced gastric cancer [19].Currently, inhibitors targeting PARP have demonstrated promising results in the treatment of various malignancies, including ovarian cancer [20], prostate cancer [21], endometrial cancer [22], among others. Thus, identifying new prognostic factors other than, for example, pathological stage to accurately identify patients at higher risk of recurrence may improve our accuracy in determining recurrence risk [23], similar to PARP9.A comprehensive exploration of the oncogenic mechanisms associated with PARP in GC may provide valuable insights for molecular interventions in GC therapy.

PARP family proteins play a crucial role in DNA repair and apoptosis [24]. Although PARP9 lacks enzymatic activity typical of other PARPs, its aberrant expression is linked to metastasis and drug resistance in various malignant tumors [15–17]. This study investigates the mechanism by which PARP9 activates the MAPK pathway through SOX6 to enhance the malignant behavior of GC. Initially, an online database was utilized to assess mRNA expression levels of PARP9 across different tumor types, followed by an analysis of its expression in TCGA-STAD, ultimately revealing that elevated PARP9 levels correlate with poor prognosis in GC patients. Subsequently, we knocked down PARP9 in GC cells, results from CCK8 assays, colony formation tests, migration studies, and invasion experiments demonstrated that PARP9 knockdown significantly inhibited cell growth while inducing apoptosis and increasing DNA damage. In contrast, overexpression of PARP9 markedly promoted cell proliferation and colony formation, further substantiating its oncogenic role. These findings reinforce the hypothesis that PARP9 functions as an oncogene in GC, positioning it as a promising novel therapeutic target.

To gain a deeper understanding of the tumorigenic activity of PARP9, we conducted RNA sequencing, which revealed that alterations in PARP9 resulted in the upregulation or downregulation of numerous target genes. Gene Set Enrichment Analysis (GSEA) indicated that these genes were associated with processes such as aging, DNA repair, recombination repair, reverse transcription, and cellular activities related to the cell cycle. Additionally, focal adhesion was found to be linked to apoptosis. These correlations align with the characteristics of PARP family genes previously described. Subsequently, we investigated potential pathways involved in the pathogenesis associated with PARP9; our findings demonstrated that the MAPK signaling pathway exhibited the highest level of enrichment.

The MAPK signaling pathway is integral to the regulation of signal transduction mechanisms that are ubiquitous across all biological processes, with abnormalities frequently observed in various cancer [25]. Notably, the aberrant activation of the MAPK pathway primarily arises from mutations in RAS and RAF proteins, which lead to constitutive activation of the extracellular signal-regulated kinase 1/2 (ERK1/2) pathway [26]. Previous studies have demonstrated that HP infection can activate both PI3K/AKT and MAPK signaling pathways in gastric epithelial cells, prompting their transformation into tumor cells through differentiation [27]. Alterations within the MAPK pathway have also been identified in different subtypes of GC, particularly involving CDH1 mutations that induce activation of the MAPK pathway [28]. Recent research has further illustrated that several distinct oncogenes facilitate the onset and progression of GC via MAPK pathway [29–31]. Collectively, these findings underscore the critical role of MAPK in GC progression. Our experimental results similarly indicate that PARP9 enhances proliferation, invasion, and migration of GC cells through modulation of the MAPK signaling pathway.

SRY (sex-determining region Y)-box 6 is a member of the SOX gene family. The SOX protein functions as a transcription factor, regulating the expression of multiple genes and participating in various biological developmental processes [32, 33]. SOX transcription factors are crucial in cancer progression, encompassing tumorigenesis, alterations in the tumor microenvironment, and metastasis. Certain SOX proteins have emerged as potential molecular markers for cancer prognosis and are considered promising therapeutic targets [34]. Notably, SOX6 has been shown to exert a influence in cervical cancer, breast cancer, and gastric cancer [35–37], while our study revealed that SOX6 levels decreased with diminishing PARP9 expression. This suggests a possible correlation between PARP9 and SOX6; we further investigated their interaction using co-immunoprecipitation (COIP) techniques. Response experiments confirmed that overexpression of SOX6 in AGS cells lacking PARP9 resulted in upregulation of apoptosis-related proteins and anti-DNA damage-associated proteins compared to those with only PARP9 knockout. Additionally, numerous studies have corroborated the association between SOX6 and MAPK signaling pathways [35, 38]. At present, the relationship between SOX6 and gastric cancer remains undetermined. Therefore, in our subsequent studies, more attention should be paid to SOX6 to better explore the pathogenic mechanism of PARP9 in GC.

Certainly, our study has several limitations. Firstly, there is an insufficient amount of in vivo study data. Secondly, the mechanistic analysis of SOX6 lacks depth. Lastly, we have not validated the clinical significance due to a lack of proprietary data.

In summary, we identified a positive correlation between PARP9 and SOX6. Furthermore, PARP9 positively regulates the anti-apoptotic properties and resistance to DNA damage in GC cells via the MAPK pathway, ultimately promoting drug resistance among these cells. Inhibiting PARP9 may represent a viable molecular therapeutic target.

## Supporting information

S1 File All raw images of western blot in this manuscript. (PDF)
