# Supplementary figures and images for: The dysregulation of PARP9 expression is linked to apoptosis and DNA damage in gastric cancer cells
Source: PLoS One. 2024 Dec 31;19(12):e0316476. doi: 10.1371/journal.pone.0316476 (PMC11687892; doi:10.1371/journal.pone.0316476)

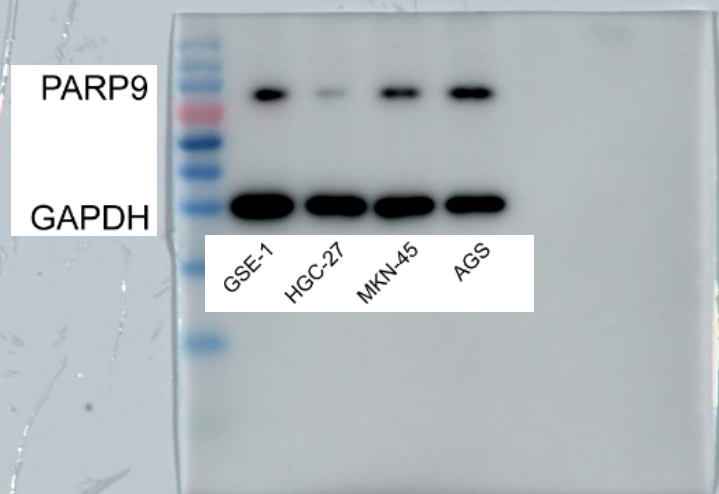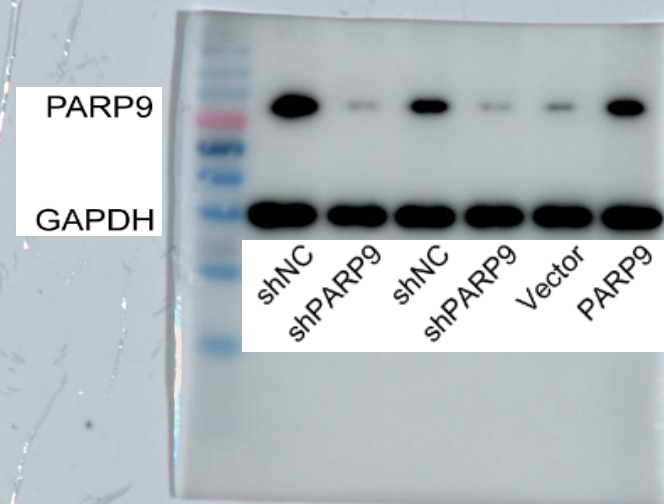

actin  
Caspase-3

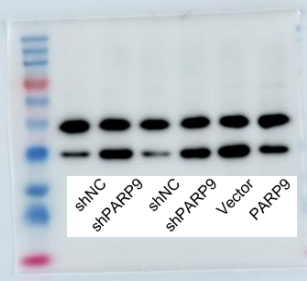

actin  
Bcl-2

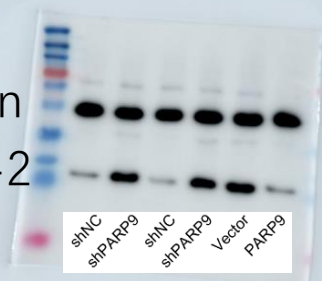

actin  
BAX

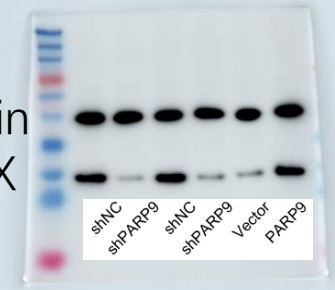

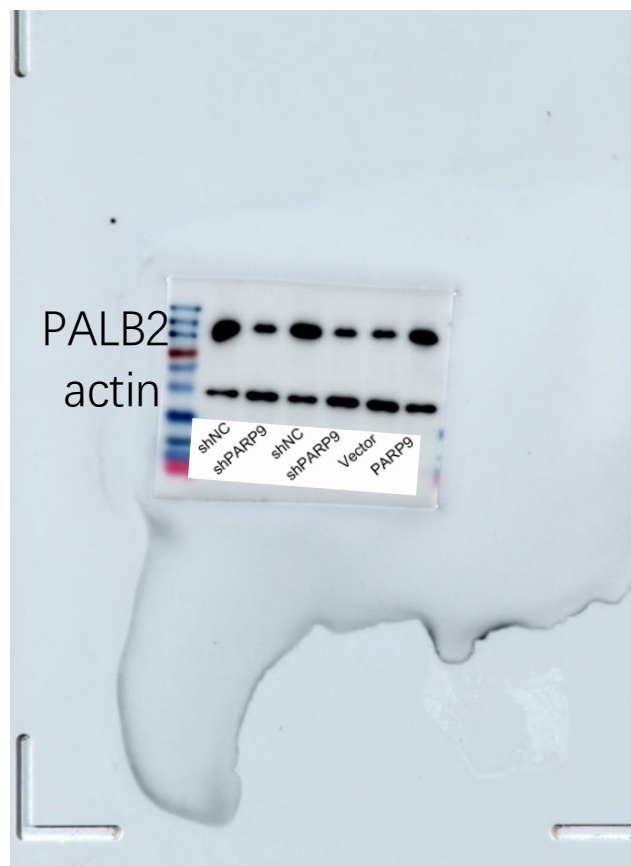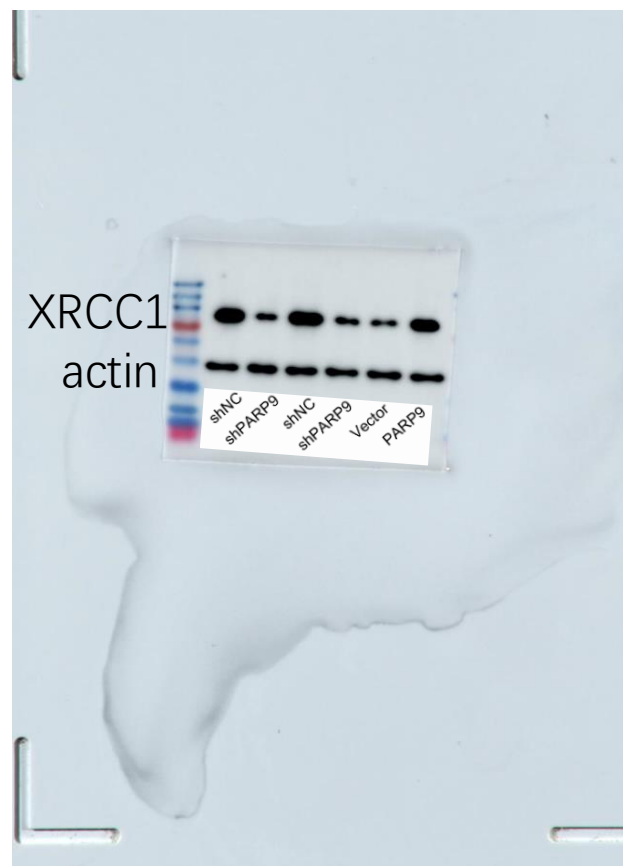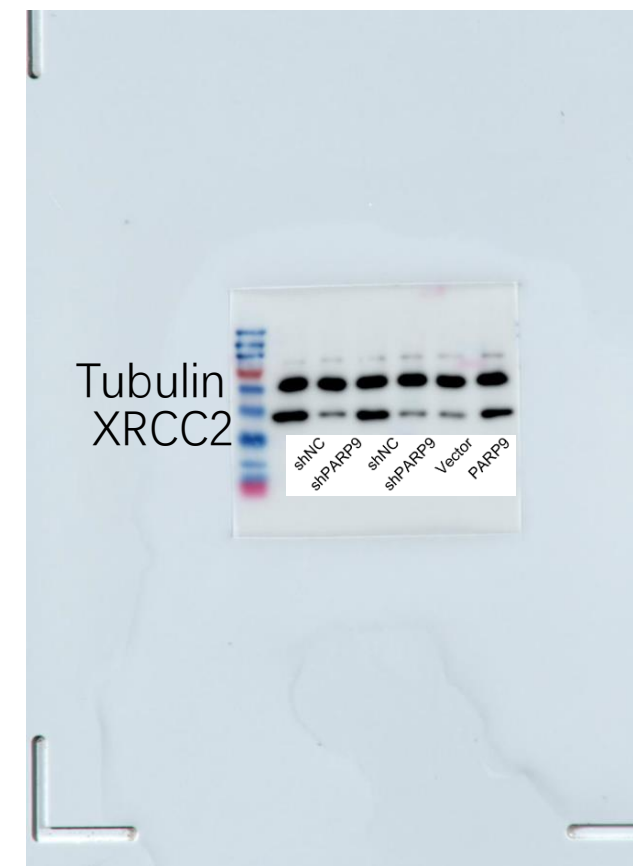

ERK1/2  
GAPDH

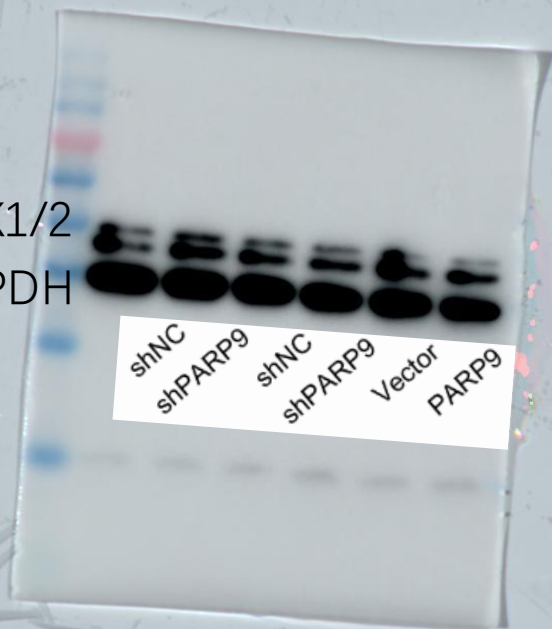

p38  
GAPDH

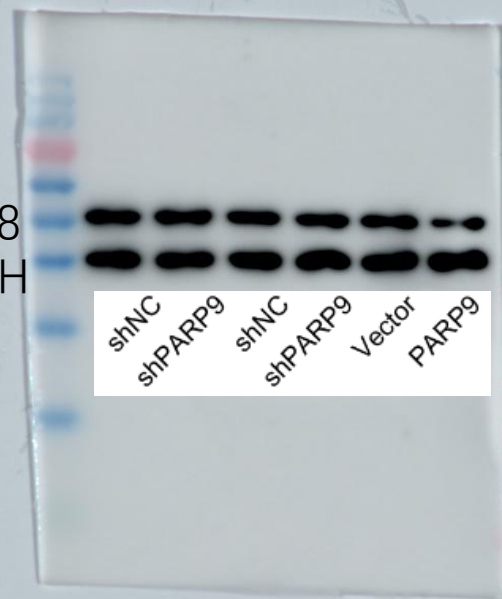

P-ERK1/2  
GAPDH

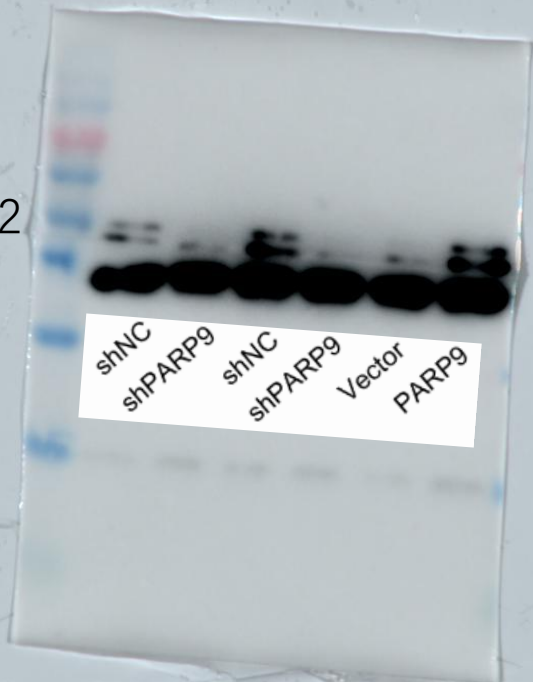

P-p38  
GAPDH

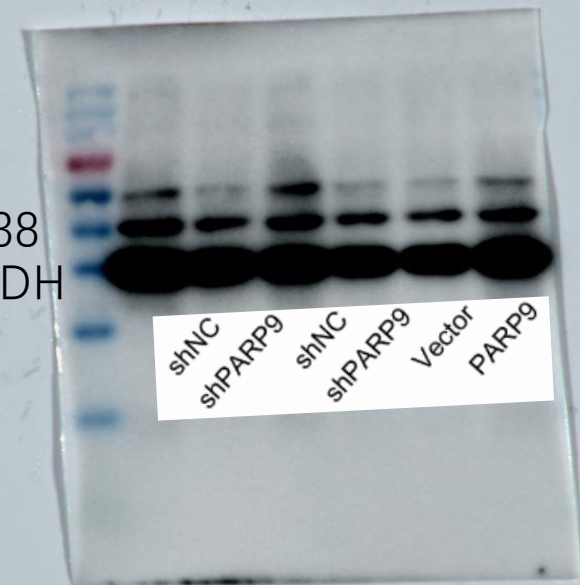

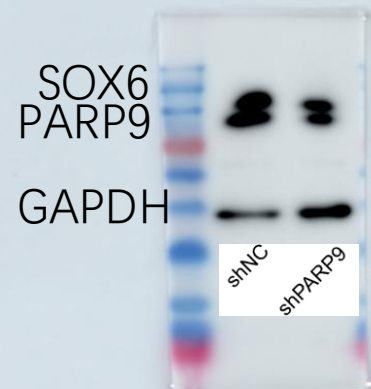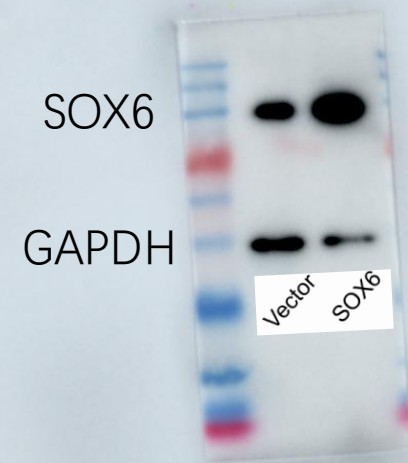

PARP9

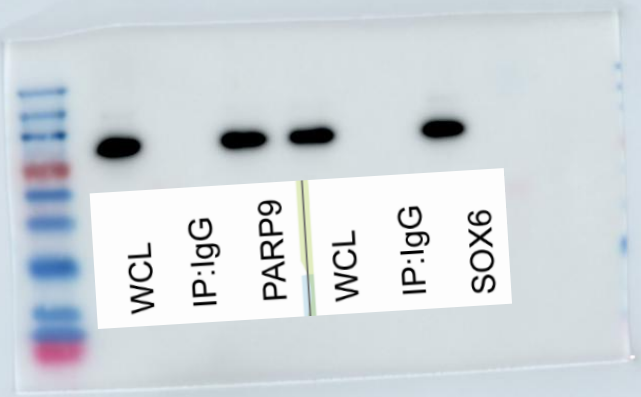

SOX6

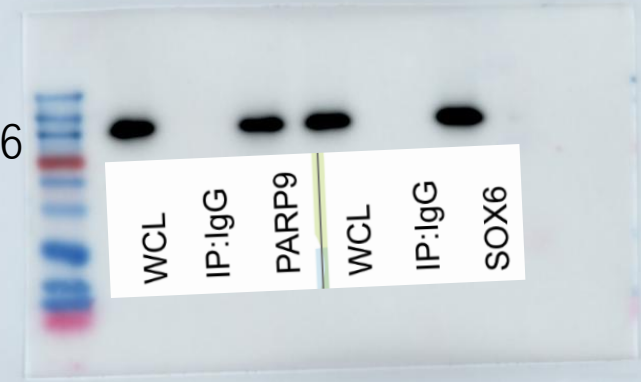

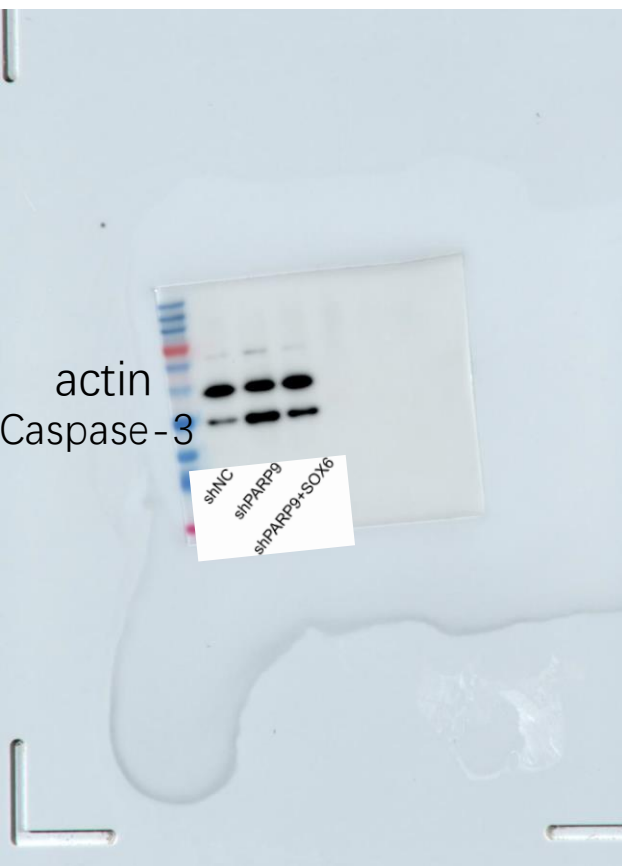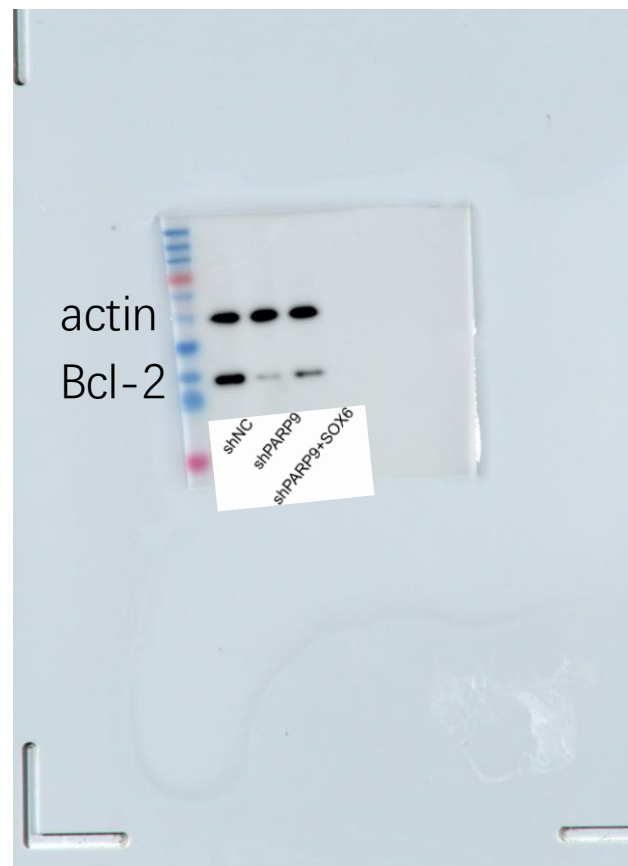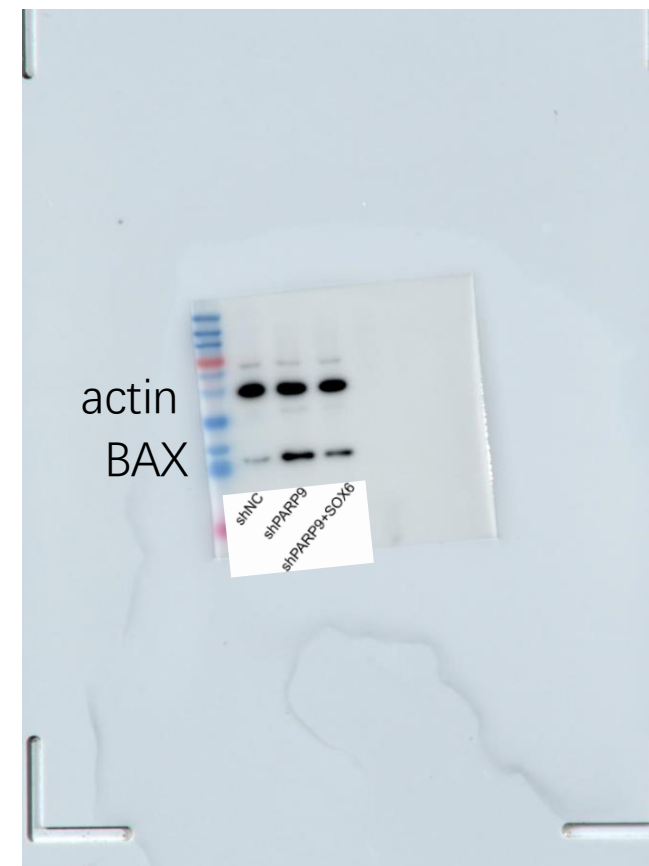

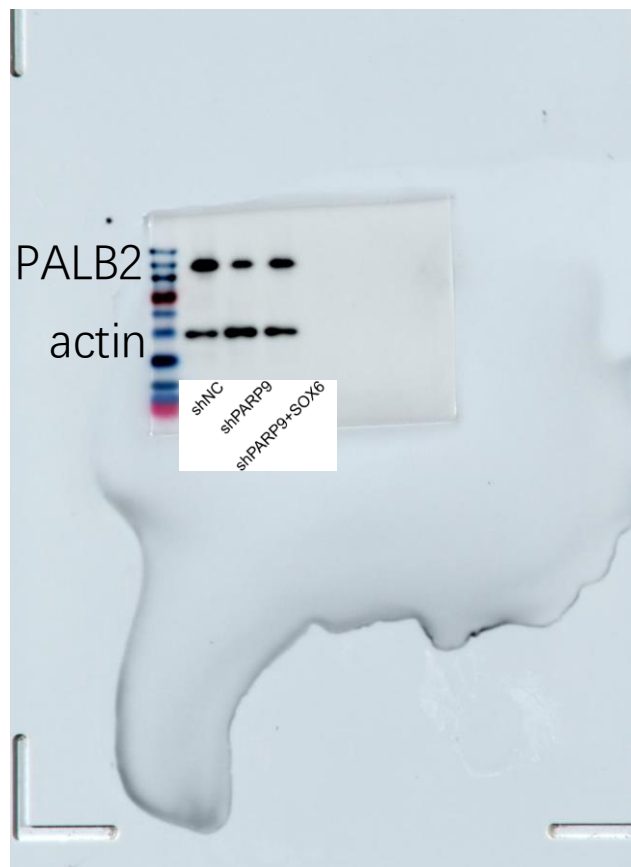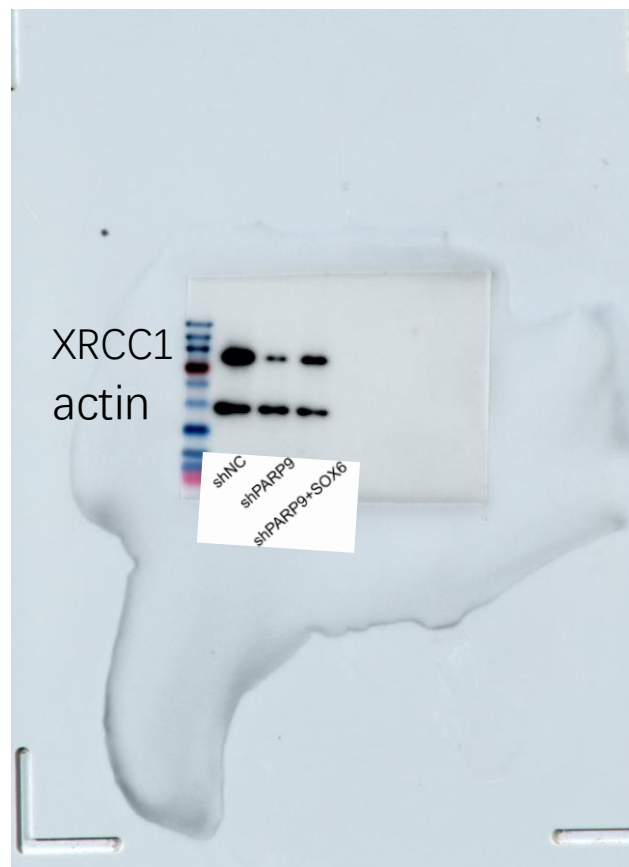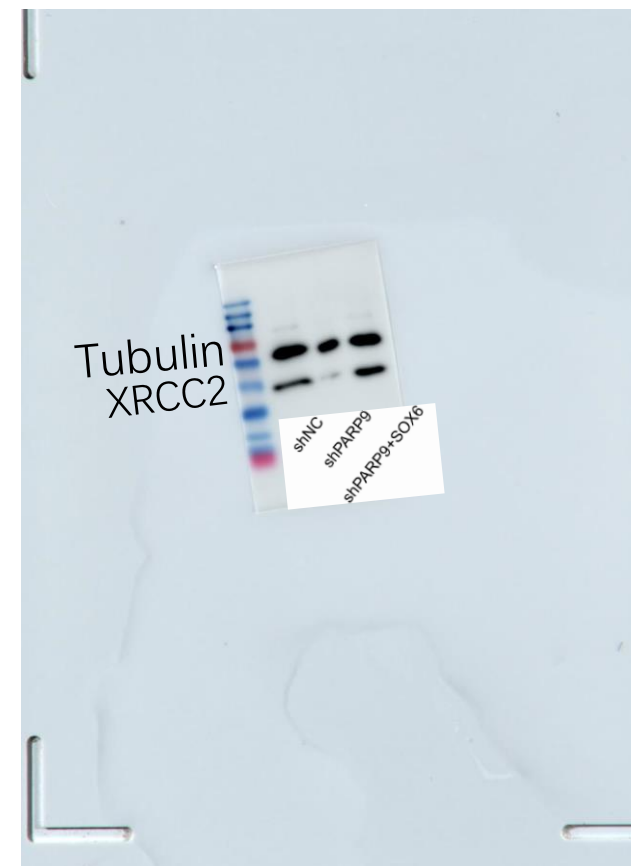

Supplement: S1 File — (PDF) [file pone.0316476.s001.pdf]
